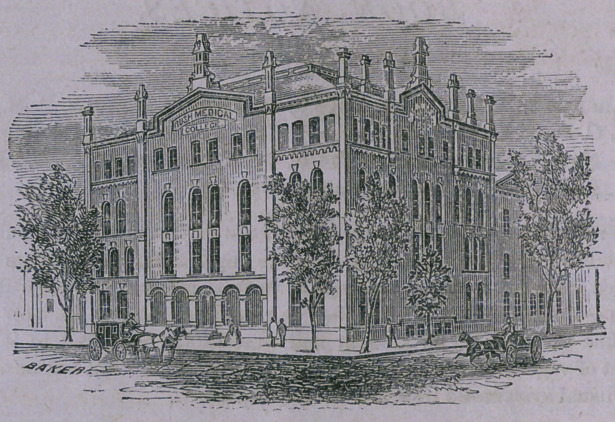# Rush Medical College

**Published:** 1868-06-01

**Authors:** 


### RUSH MEDICAL COLLEGE,

### CHICAGO.

---

*Annual Announcement and Circular for* 1868-9.

---

THE Introductory Lecture to the Twenty-Sixth Annual Course will be given in the lower lecture-room of the College, on Wednesday Evening, September 30th, by PROFESSOR POWELL, and the regular lectures will commence on the following morning, and continue eighteen weeks.

The control of the College is vested in the following:

### *Board of Trustees.*

HON. W. B. OGDEN, - - - - PRESIDENT.
HON. GRANT GOODRICH, - - SECRETARY.
HON. MARK SKINNER, - - - TREASURER.

E. S. KIMBERLY, M.D.

L. C. P. FREER, ESQ.

HON. HUGH T. DICKEY.

W. L. NEWBERRY, ESQ.

GEORGE W. SNOW, ESQ.

HON. N. B. JUDD.

J. W. FREER, M.D.

EPHRAIM INGALS, M.D.

HON. RICHARD J. OGLESBY, *Governor.*

HON. FRANKLIN CORWIN, *Speaker H. R.*

JAMES V. Z. BLANEY, M.D., *Pres. College.*

} *Ex-Officio.*

### *Faculty.*

J. V. Z. BLANEY, A.M., M.D., President,
*Prof. of Chemistry and Pharmacy*, 45 Clark Street.
JOS. W. FREER, M.D.,
*Prof. of Physiology and Microscopic Anatomy*, College.
J. ADAMS ALLEN, M.D., LL.D.,
*Prof. of Principles and Practice of Medicine*, 71 Dearborn Street.
E. INGALS, M.D., Treasurer,
*Prof. of Materia Medica and Medical Jurisprudence*, 190 Clark Street.
DELASKIE MILLER, M.D., Secretary,
*Prof. of Obstetrics and Diseases of Women and Children*, 518 Wabash Ave.
R. L. REA, M.D.
*Prof. of Anatomy*, 119 Clark Street.
MOSES GUNN, A.M., M.D.,
*Prof. of Principles and Practice of Surgery and Clinical Surgery*, College.
EDWIN POWELL, M.D.,
*Prof. Military Surgery and Surgical Anatomy*, 45 Clark Street.
JOSEPH P. ROSS, M.D.,
*Prof. Clinical Medicine and Diseases of the Chest*, 85 Washington St.
EDWIN L. HOLMES, M.D.,
*Lecturer on Diseases of the Eye and Ear*, 169 Dearborn Street.
CHARLES T. PARKES, M.D.,
*Demonstrator*, 43 North Clark Street.
WILLIAM LITTLE, M.D.,
*Curator of the Museum and Prosector of Surgery*, College.

Charles Keil, *Janitor*, at the College.

### *College Building.*

The College building, situated on the corner of Dearborn and Indiana Streets, contains two large Lecture-rooms, each having 625 numbered seats, a spacious Laboratory, Museum, and Dissecting-room,—the latter, thirty-eight by eighty feet in size, thoroughly ventilated, lighted by side and skylights, and abundantly supplied with gas and water, containing fifty tables, and private closets for the wardrobes, books and instruments of each class, is unquestionably unequaled for its purposes. The whole building is warmed by steam, by which a uniform and agreeable temperature is secured in all its parts. Several of the Faculty have their offices in the building, affording every requisite facility for students to secure such information as they may from time to time desire. The experience of the last session fully attests the perfect success of the appliances for both heating and ventilation. Not a single case of severe sickness occurred in the large elass assembled. Contrasting this with the results of previous years, it is clear that, aside from mere size, very important hygienic improvements have been secured in the new structure. The Faculty are fully sustained in the statement that the assembling class will be welcomed to the finest and most commodious Medical College building in the world.

All members of the profession are invited to call and survey the building and its advantages at any time.

### *Clinics.*

The Clinical advantages of the College are ample and varied.

A daily clinic will be given at the Marine Hospital. Prof. GUNN and Dr. E. C. ROGERS, Surgeon in Charge, will lecture upon Clinical Surgery, and Dr. WILLIAM C. LYMAN, (late Surgeon U.S.N., now Resident Physician of the Hospital,) upon Diseases of the Chest.

Four Clinics each week will be given at the County Hospital. It is a commodious building, containing about three hundred beds, and being attended by an able corps of Physicians and Surgeons, will, in addition to the Marine Hospital, the Chicago Eye and Ear Infirmary, and the College Dispensary, give very abundant clinical material for the observation of the class. During the year there were seventy-three autopsies at the County Hospital, which afforded to the class in attendance excellent opportunity for the study of morbid anatomy. The field for study of diseases of the chest, is probably not excelled in this country.

The connection of Profs. POWELL and ROSS with the Hospital is sufficient guarantee that every facility will be extended to students to secure the highest advantage from Clinical teaching.

The Lecturer on Diseases of the Eye and Ear, in addition to his course at the College, will give Clinical instruction twice a week, at the Chicago Charitable Eye and Ear Infirmary, which has been enlarged and furnished with all the conveniences necessary for patients with diseases of these organs. Students will have an excellent opportunity of studying diseases of the eye, and of witnessing many operations in ophthalmic surgery.

Saturday of each week will be devoted to surgical operations and Clinical instruction in the College, at which time indigent persons, who require advice or surgical operations, are attended gratuitously.

The advantages derived from these *cliniques* consist in the opportunity of witnessing a great variety of surgical operations and numerous medical cases, and the treatment proper for each.

Since the re-opening of the Dispensary at the College, the number of patients in daily attendance has largely increased, furnishing opportunities for observation unsurpassed in this country. As noted in another place, the importance of securing the best possible results from the abundant clinical material has been recognized by the Board of Trustees, in the establishment of a new chair having especial supervision of this department, and intended to develop its advantages to the utmost.

### *Directions to Students.*

Students will sign the Matriculation List, and obtain their tickets of the Treasurer, Prof. INGALS, at his office, 190 Clark Street; and to give opportunity for this, the office will be open from 8 o'clock A.M. to 5 o'clock P.M., on and after Monday, September 28th. Students may select their seats in the lecture rooms when they take their tickets. The Janitor may be seen at his residence in the College building, and will aid in obtaining boarding places, rooms, etc. For any special information, students may call on any member of the Faculty. For circular, address the Secretary, Prof. DELASKIE MILLER, 518 Wabash Avenue.

### *Graduation.*

The following are the requirements for the Degree of Doctor of Medicine, viz.:

1st. The candidate must be twenty-one years of age, and give satisfactory evidence of possessing a good moral character.

2nd. He must have pursued the study of medicine three years, and attended at least two courses of Lectures. one of which must have been in this Institution.

3rd. He must have attended Clinical Instruction during, at least, one College term.

4th. He must have pursued the study of Practical Anatomy, under the direction of the Demonstrator, and to the extent required by the rules of the College.

5th. He must notify the Secretary of the Faculty of his intention to become a candidate, and deposit the amount of the Graduation Fee with the Treasurer on or before the 20th day of January. In case the candidate fails to graduate, the fee will be returned to him.

6th. Every candidate must undergo a full and satisfactory examination on all branches taught in the College.

7th. Graduates of other respectable schools of medicine will be entitled to an *ad eundem* degree, by passing a satisfactory examination, paying the graduation fee, and giving evidence of a good moral and professional character.

### *Books of Reference.*

Students will find a good assortment of medical books and surgical instruments in this city. The following books of reference, among others, are recommended:

*Chemistry.*—Fownes, Brande and Taylor, Wells.

*Anatomy.*—Gray, Wilson.

*Physiology.*—Todd and Bowman, Flint, Dalton, Draper.

*Materia Medica.*—Waring, Stillé, Wood, U. S. Dispensatory, H. C. Wood's Abridgment of Pereira.

*Medical Jurisprudence.*—Taylor, Beck, Wharton, and Stillé.

*Obstetrics.*—Hodge, Cazeau, Bedford, Churchill.

*Diseases of Women.*—Scanzoni, West, Thomas.

*Diseases of Children.*—Condie, West, Tanner, Bouchut.

*Surgery and Surgical Pathology.*—Erichsen, Chelius, Druitt, Gross, Paget.

*Practice of Medicine.*—Flint, Aitken, Wood, Watson. Bennett's Clin. Med; Flint, or Walsh, on the Heart; Walshe on the Lungs; Habershon on the Alimentary Canal; Wilson on the Skin; Da Costa, or Barclay, on Diagnosis; Chambers, Rokitansky, Jones and Sieveking; Hammond, Military Hygiene.

*Surgical Anatomy.*—Maclise.

*Microscopic Anatomy.*—Todd and Bowman, Queckett.

### *Fees.*

| | |
|---|---|
| Lecture Fees for the Course | $50 00 |
| Matriculation Fee | 5 00 |
| Dissecting Ticket | 5 00 |
| Hospital Tickets | 5 00 |
| Graduation Fee | 25 00 |

From Alumni of this and other respectable Medical Colleges, the Matriculation Fee only will be required.

### *Board and Rooms.*

Good board, with rooms and all the usual accommodations, can be obtained at as reasonable rates as in any other city. By associating in clubs, students may supply themselves with good accommodations at a material reduction from ordinary rates.

### *Spring and Summer Instruction.*

Under the direction of the Faculty, a Spring and Summer Course is annually conducted, consisting of lectures, recitations, and clinical observations, at the Hospitals and College Dispensary. It is not intended that it shall be in lieu of a regular course, but is established to afford greater facilities to students desiring to remain in the city during summer for the benefit of its clinical advantages.

The outline of this course will be published before the close of the next session.

### *School of Chemistry.*

Since the close of the last session, the spacious Laboratory of the College has been fitted up for instruction in *practical* Chemistry. Students are received at any time, and, if desired, can have the privilege of working at the tables, under the immediate direction of the Professor of Chemistry. Several courses are given, and students can enter at any time, upon any one of the courses It is, however, desirable that classes should be formed, the members of which will progress together Special instruction is given in the several departments of Chemistry as follows: Rudimentary Chemistry, Qualitative Analysis, Quantitative Analysis, Practical Toxicology, Chemistry of the Urine, and Physiological Chemistry, Instruction in Practical Metallurgy and Assaying, and Chemistry of Arts and Manufactures, will be given to those who desire it.

Those desirous of the special advantages of this branch of study, and wishing further information, may address Prof. J. V. Z. BLANEY, at Rush Medical College.

### *Miscellaneous.*

The means of illustration in the several departments are ample and constantly increasing.

In PRACTICAL ANATOMY, arrangements have been perfected which will secure an abundance and cheapness of *materièl* at reasonable rates.

During the coming session, Prof. POWELL will engage in the active duties of his chair.

The appointment of Prof. ROSS to the newly-created chair of CLINICAL MEDICINE AND DISEASES OF THE CHEST, will commend itself to the judgment of the profession, as of great importance. His especial duty will be to develop the clinical advantages of the College. An earnest, efficient worker, frequently honored by the suffrages of the profession, officially connected with the hospitals of the city, there is no doubt that his selection will meet with general favor.

In MATERIA MEDICA, a complete and entirely new cabinet of specimens has been procured, together with a full set of colored plates of medicinal plants.

In OBSTETRICS, in addition to the usual didactic instruction, cases of labor will be placed under the charge of advanced students. During the last session, a large number of students enjoyed such opportunities.

Prof. FREER is again visiting Europe, but will return to give his course, bringing with him all the advantages to be gained by critical observation in the Old World. His instruction will be fully illustrated by his usual *vivisections* and experiments.

Large accessions have been received to the MUSEUM, and the thanks of the Faculty are tendered to friends of the College for liberal donations of valuable specimens.

### GRADUATES OF RUSH MEDICAL COLLEGE,

### SINCE ITS ORGANIZATION.

SESSION 1843–44.

Wm. Butterfield

Thomas P. Whipple }

John McLean } *Honorary Degree.*

1844–45.

Alfred E. Ames
William Fosdick
Edwin R. Long
Ira E. Oatman
Josiah B. Herrick
Almon W. King
Samuel W. Ritchey
Nehemiah Sherman
Stephen Munroe, Jr.
Isaac Watts Garvin
Arnold H. Neadham

1845–46.

Elwood Andrew
J. Herman Bird
Daniel K. Hays
James M. Higby
Newton P. Holden
Alexander B. Malcolm
Cicero Robbe
Halsey Rosenkrans
Robert Scott
William W. Welch

W. G. Montgomery, M.D., *Honorary.*

1846–47.

H. I. E. Balch
S. A. Barry
I. R. Bradway
Joseph Blount
M. B. Elgin
A. V. Gilbert
Fred. E. Hagemann
H. P. Hernes
Ephraim Ingalls
Philip Kirwin
Leonard L. Lake
Lafayette W. Lovell
E. A. Gilbert
Wesley Pierce
Isaac Snyder
James F. Saunders
J. C. Leary
David J. Peck
J. E. McGirr

Samuel Grimes, M.D., *Honorary.*

1847–48.

Daniel M. Camerer
W. Chamberlain
J. A. Clark
A. B. Crawford
Milton D. Darnell
Uri P. Golliday
R. S. Hawley
I. C. H. Hobbs
E. G. Hough
G. J. Huey
Ambrose Jones
C. W. Knott
J. C. Lovejoy
Sample Loftin
William Matthews
Thomas C. Moor
I. H. McNutt
John Newton
John Nutt
O. C. Otis
I. G. Osborn
J. Pearson
A. Reynolds
W. W. Sedgewick
Warren M. Sweetland
R. R. Stone
James P. Tucker
C. C. Warner
L. W. Warren
Charles Ware

E. S. Kimberly, *Honorary.*

1848–49.

Alfred W. Armstrong
William W. Cunnerly
Asa Clark
Harvey Cutler
Joseph W. Freer
Charles C. Garrett
Israel G. Harlan
George M. Huggans
Calvin B. Lake
Robert Pennel Lamb
Orrin T. Maxson
Peter B. McKay
Edwin G. Meek
Gideon C. Paramore
James C. Patterson
Charles H. Richings
John H. Warren
Jerome F. Weeks

Dr. Thomas Hall, Dr. James H. Budd, *Honorary.*

1849–50.

Joseph L. Anderson
Clay Brown
Thomas D. Brown
Cyrus G. Blood
Henry F. Brown
Willard F. Coleman
Kimball Favor
Edward J. French
John Gregory
Isaiah P Hamilton
S. Rush Haven
George Higgins
Orson C. Hoyt
Alexander Hull
Franklin B. Ives
M. Tevis Klepper
Thomas G. Klepper
Charles J. Macon
Alonzo L. McArthur
Manly Miles, Jr.
Risdon C. Moore
William C. Oatman
Silas S. Parkhurst
William J. Paugh
John M. Phipps
William W. Perry
Giles P. Ransom
David Rogers
Josiah R. Snelling
John W. Spalding
Benjamin G. Stephens
Benjamin F. Stephenson
Edwin Stewart
Isaac E. Thayer
John M. Todd
Henry D. C. Tuttle
Harmon Wasson
Jas. P. Walker
George S. Wheeler
Zachariah H. Whitmire
Thomas Wilkins
William W. R. Woodbury
James R. Zearing

James S. Whitmire, M.D., *ad eundem.* Dr. E. S. Cooper, *Honorary.*

---

1850–51.

Gordon Chittock
S. L. Craig
F. W. Coolidge
J. H. Constant
G. S. Crawford
William M. Crowder
O. D. Coleman
H. C. Donaldson
C. J. Hull
J. C. Hinsey
A. M. Johnson
V. P. Kennedy
T. S. Loomis
H. E. Luther
L. D. Latimer
R. Morris
J. H. Murphey
L. A. Mease
S. R. Mason
G. C. Merrick
J. P. Porter
L. C. Pomeroy
B. O. Reynolds
William W. Sweeney
E. T. Spotswood
S. T. Trowbridge
A. M. Thorn
C. Van Doren
Edwin Wright
John Walker

James S. Russell, M.D., *ad eundem.*
Dr. James G. M. Meehan, Dr. Thompson Mead, *Honorary.*

---

1851–52.

Henry D. Adams
George W. Albin
Franklin Blades
Benjamin T. Buckley
George A. Bodenstab
G. Judson Bentley
William D. Craig
F. Marion Crouse
Alexander B. Chadwick
Theodore G. Cole
James A. Collins
Alexander De Armond
William H. Davis
John Garrison
Walter R. Godfrey
Stephen C. Gillett
William C. Hunt
Vincent L. Hurlbut
Marsena M. Hooton
William M. Hobbie
Orvis S. Johnson
Hosmer A. Johnson
Hiram C. Jones
Abram H. Knapp
Isaiah P. Lynn
Ezra M. Light
Hugh Marshall
Lewis D. Martin
M. G. Parker
J. Harrison Reeder
Dudley Rogers
A. F. St. Sure Lindsfelt
Leander D. Tompkins
Ezra Van Fossen
Edwin R. Willard
John D. Woodworth
Jeremiah Youmans

---

1852–53.

Robert F. Bennett
J. A. Breneman
D. Alphonso Colton
P. G. Corkins
William Curliss
O. D. Chapman
J. P. Cunningham
Elijah H. Drake
Hosea Davis
M. F. Gerard
Robert F. Henry
S. B. Harriman
Oliver S. Jenks
J. A. James
Warren Millar
Solon Marks
James B. Moffett
Henry Parker
H. W. Ross
John F. Starr
Henry S. Steele
Josiah Stanley
Hiram Smith
J. B. Wheaton
S. H. Whittlesey
R. Q. Wilson
Daniel Whitinger

A. D. Dwight
Robert W. Earle
James Gregory
John Phillips
James M. Proctor
Arthur Young
William M. Young

---

1853–54.

Wm. M. Avery
Albert Boomer
Washington Brenton
John W. Collver
Charles C. Cornett
Charles W. Davis
Isaac N. Davis
Joseph M. Edwards
Joseph N. B. Elliott
Hezekiah Fisk
Melancthon W. Fish
Thomas D. Fitch
William A. Hillis
Roscoe L Hale
John F Hamilton
Richard S. Hallock
Edward Hopkins
Anderson W. King
John W Lynch
William Manson
Harvey C. Morey
Henry W Mann
J. B. Morrison
R. M. McArthur
John T. Mayfield
John N. Niglas
Myron W Robbins
Simeon P. Root
Reuben Sears
William B. Swisher
George W. Slack
Thomas P. Seller
Charles D. Watson
William Watson
Enos P. Wood
David Whitmire
Stephen P. Yeomans

---

1854–55.

George A. Byrns
Jesse Barber
Lewis C. Bicknell
Horace C. Clapp
Michael R. Chadwick
Thaddeus M. Crombie
Berry W. Cooper
Hiram L. Coon
Solomon S. Clark
Jason N. Conley
Mordecai Davis
Darwin DuBois
James Evans
James Ford
Charles Gorham
George T. Goldsbury
James F Grove
Vernon Gould
Christopher Goodbrake
Thomas R. Hanna
Freeborn F. Hoyt
Alonzo L. Hutchinson
Elisha G. Horton
William H. Heller
Charles W. Jenks
Leroy H. Kennedy
John McHugh
John F. McCarthy
James C. McMurtry
Ross W. Pierce
Isaac Rice
Hugh Russell
Homer C. Rawson
Allen A. Rawson
James M. Suddath
John W. Trabue
Henry Van Meter
William Van Nuys
Hiram J. Van Winkle
Martin Wiley
Elias Wenger

---

1855–56.

Meredith C. Archer
J. Milton Barlow
Daniel Bowers
Almond C Buffam
Edward W. Boothe
David W. Carley
John W. F. Clawges
A. B. Carey
A. Jackson Crain
James L. Crain
Francis M. Constant
John E. Deming
Hamilton C. Daniels
Roswell Eaton
John J. Everhard
Edwin Gaylord
James P. Graham
William F. Green
James W. Green
William A. Gordon
Samuel Griffith
Robert Hitt
George W. Kittell
H. W. Kreider
David T. Kyner
L. L. Leeds
B. S. Lewis
D. LaCount
A. A. Lodge
D. M. Marshall
T. C. McGee
Z. H. Madden
B. G. Neal
W. H. Phillips
J. R. Robson
Bailey Rogers
F. Ronalds
Lee Smith
Joseph Williamson
Horace Wardner
R. Winton

J. Henderson, M.D., *ad eundem*. Dr. M. M. Latta, *Honorary*.

---

1856–57.

A. W. Adair
J. S. Bowen
M. H. Bonnell
D. C. Bennett
E. F. Hubbard
A. M. D. Hughes
A. L. Kimber
J. C. Lowrie
L. H. Smith
D. H. Spickler
J. H. Tyler
J. P. Terrell

J. F. Cravens
L. D. Dunn
T. B. Dever
T D. Fisher
T. A. Graham
Lafayette H. Gray
Samuel Higinbothem
W. M. Hall
C. Hill
Charles Hamill
J. J. Luke
J. T. Miller
J. F. Marsh
E. McAferty
J. McCleary
J. B. Paul
Edwin Powell
J. L. Phillips
N. O. Pearson
T. J. Shreves
S. L. Urmston
W F. Vermillion
B. Wilson
B. F. White
P. J. Wardner
G. W. Wilkinson
E. A. Wilcox
B. Woodward
F. W. White

J. W. York, M.D., *ad eundem.*
Dr. William Long, Dr. H. Noble, *Honorary.*

1857–58.

L. B. Brown
L. Brookhart
R. C. Black
Freeman Clark
P. Corcoran
S. B. Davis
Benjamin Durham
J. B. Earl
C. N. Ellinwood
W. B. Harl
Allen Heavenridge
J. N. Green
J. D. Gray
T. C. Jennings
B F. Keith
Charles J. Keegan
Willis May
W. L. May
A. J. Miller
D. B. Montgomery
John O'Connor
O. B. Ormsby
J. T. Pearman
J. L. Patten
J. S. Pashley
B. F. Ross
W. H. Rockwell
J. Slack
William Somers
C. V Snow
L. D. Smedley
Benjamin F. Swofford
Owen Wright
J. D. Webster
J. B. Wilson
Thomas Winston
Eli York

Solomon Davis, M.D., Waldo W. Lake, M.D., *Honorary.*

1858–59.

L. Grant Armstrong
E. H. Ayres
Benjamin W. Bristow
A. M. Blackman
John A. Cook
George W. Corey
J. R. Conklin
N. M. Douthitt
E. C. Dickinson
John H. Farrell
Richard Hull
William C. Hopwood
Blixton Harris
William L. Kreider
J. W. W. Lawrence
W. H. Lyford
Lafayette Lake
R. McGee
F. Mason
Samuel McNair
J. R. Pearce
W. E. Peters
E. O. F Roler
E. A. Steele
P. R. Slingsley
A. B. Taylor
Myron Underwood
E. L. Welling
R. F. Williams
J. H. Wiley
J. F. Williams

J. Drake Harper, M.D., *ad eundem.* S. M. Mitchell, *Honorary.*

1859–60.

Orson B. Adams
John J. M. Angear
John T. Billington
Frederic Bartels
John B. Baker
Edward L. H. Barry
Hiram Carnahan
Henry Durham
B. I. Dunn
John Dancer
Rufus M. Elliott
John E. Ennis
John B. Felker
A. M. Golliday
Jethro N. Hatch
Daniel Kirkpatrick
Thomas I. Fritz
Leigh R. Holmead
Milton N. Isaac
William Irwin
Hiram C. Luce
John McDamron
Percy McAlpin
Philip Matthei
Wm. F. Osborn
George W. Richards
Edward Thomas
James Thompson
Vincent S. Thompson
J. S. Underwood
Wm. V. Wiles
Samuel N. Sheldon
C. M. Smith
Robert B. Ray
James F. Spain

Dr. Calvin Wheeler, *Honorary.*

1860–61.

| | | |
|---|---|---|
| Willford Bates | Wm. B. Graham | Richard E. McVey |
| Charles Bunce | Henry J. Herrick | John Murphy |
| Allen S. Barndt | Zenas P. Hanson | Samuel C. Owen |
| Wm. C. Brown | Clinton D. Henton | Allen M. Pierce |
| Sidney S. Buck | Ezekiel Keith | Henry V. Passage |
| Benjamin H. Bradshaw | John T. Keables | Madison Reece |
| Henry S. Blood | Enoch W. Keegan | E. Fred. Russell |
| Elijah A. Clark | Abner D. Kimball | Theodore W. Stull |
| Daniel M. Cool | Robert M. Lackey | Edward P. Talbott |
| Thomas J. Dunn | Z. James McMaster | Charles B. Tompkins |
| Edward C. De Forest | James M. Mayfield | Israel B. Washburn |
| Morton M Eaton | Henry H. Maynard | O. G. Walker |
| George Egbert | | |

Dr. Robert C. Hamill, Dr. Theodore Hoffman, *Honorary.*

1861–62.

| | | |
|---|---|---|
| Albert A. Ames | Thomas G. Drake | William Rush Patton |
| Charles E. Allen | James B. Farrington | Holland W. Richardson |
| Stephen G. Armstrong | A. Z. Huggins | William R. Russell |
| George W. Beggs | Jacob H. Houser | Charles M. Richmond |
| Aurelius T. Bartlett | Riley B. Hayden | Robert E. Stevenson |
| Leonard L. Bennett | Jacob M. Hagey | Samuel B. Ten Broeck |
| James Brown | Clark E. Loomis | I. Allen Torrey |
| Elijah W. Boyles | I. Meek Lanning | Alfred H. Whipple |
| William L. Cuthbert | George J. Monroe | D. Bishop Wren |
| J. Griffin Conley | William Meacher | John A. Ward |
| William D. Carter | William McKnight | Egbert H. Winston |
| Samuel M. Dunn | Fordyce R. Millard | |

J. C. Taggart, M.D., *Honorary.*

1862–63.

| | | |
|---|---|---|
| Gordon Andrews | Daniel C. Jones | Wesley Phillips |
| Charles F. Barnett | Hiram M. Keyser | Byron G. Pierce |
| Ela L. Bliss | Charles B. Kendall | William C. Piatt |
| E. Bishop | James Kelly | John M. Rankin |
| Frederick W. Byers | Edward E. Lynn | James I. Ransom |
| James Cunningham | Charles F Little | Lemuel H. Rogers |
| Philo W. Chase | G. Allen Lamb | Fernando C. Robinson |
| John W. Dean | James Muncey | Lewis H. Skaggs |
| William B. Dunkle | George C. McFarland | John W. Saucerman |
| Charles F. Dilly | Frank C. Mehler | Abram L. Small |
| Charles F. Elder | James H. McNeil | W. H. Smith |
| Francis A. Emmons | Thomas H. Montgomery | H. W. Sigworth |
| Uriah B. Ferris | John McLean | William Scott |
| Stephen N. Fish | Samuel L. Marston | William H. Tompkins |
| William M. Gregory | L. Pitt Y. McCoy | Pembroke R. Thombs |
| Harrison H. Guthrie | Elmer Nichols | John Ll. Williams |
| Myron Hopkins | J. Copp Noyes | William T. Wilson |
| Pryer J. Herman | Cornelius O'Brien | James A. Williams |
| George F. Heideman | Jacob W. Ogle | John Zahn |
| Samuel G. Irwin | | |

1863–64.

| | | |
|---|---|---|
| Frank B. Adkins | Lewis H. Goodwin | Jabez H. Moses |
| Harrison Akely | J. J. Gulick | Alexander P. Nelson |
| Orlenzer Allen | J. Milton Hiatt | Eugene L. Nelson |

Samuel J. Avery
Lyman F. Babcock
Charles M. Babcock
A. J. Bacon
S. K Barclay
G. Frank Beasley
George R. Bibb
William T. Bradbury
Charles A. Bucher
Spencer Byrn
Frank D. Cass
F. Marion Cassell
Ellston Chamberlin
James E. Coakley
Ephraim Dayton
James W. Dora
T. B. Dora
Franklin Eells
J. Wesley Egbert
F. Edwin English
J. B. Fares
Horace Gaylord
E. T. Glasener
J. A. Goldsbury
Robert L. Hill
H. C. Hollingsworth
Frank A. Jordon
Erwin L Jones
Augustus P. C. Jones
I. C. Johnson
George N. Jennings
John J. Kelly
Leslie E. Keely
Robert S. Kelso
John R. Kerrell
A. H. Kinnear
L. J. M. Kords
Bartlett Larimer
Gilbert B. Lester
Timothy T. Linn
Lorenzo D. Lowell
J. Ellis Lyons
S. B. McGlumphy
Peter S. McDonald
Samuel Mendenhall
Henry A. Mix
Martin E. Munger
James A. Monroe
J. N. O'Brien
Roswell R. Palmer
G. Hial Peebles
Edward H. Price
Charles M. Richardson
Philip Shaffer
George W. Schuchard
William A. Smith
J. M. Still
J. Dwight Stillman
John M. Swift
John W. Thayer
Joel T. Tevis
Marvin Waterhouse
John M. West
William F. Welsh
J. A. Williams
James M. Watkins
G. D. Winch
Samuel Wilson
Charles A. White
Orlando S. Wood
Titus P. Yerkes

Chas. White, M.D., Frederick S. C. Grayston, M.D., *ad eundem.*

1864–65.

W. R. Adair
J. Madison C. Adams
Henry Allen
R. M. Allen
W. C. Baird
Braxton Baker
Zopher Ball
John Becker
Newton Baker
C. R. Blackall
E. J. Bond
D. W. Bosley
W. E. Bowman
James G. Boardman
J. W. Brown
W. H. Bright
J. G. Blanchard
C. H. Brunk
C. H. Carlisle
E. P. Catlin
W. E. Chamberlin
H. F. Chesbrough
Frederick Cole
Samuel Cole, Jr.
H. N. Clark
J. L. Congdon
J. Cooper
John Cotton
Clinton Cushing
M. Morton Dowler, Jr.
Andrew J. Eidson
Samuel S. Elder
Smith T. Ferguson
S. A. Ferrin
Henry A. Folger
O. D. Ford
J. H. Foster
Samuel Galloway
H. T. Godfrey
R. Romanta Gaskill
J. Thomas Hale
J. M. Harrah
Thomas C. Hance
A. P. Herndon
Wm. H. Hess
Smith H. Hess
J. W. Herdman
Francis M. Hiett
H. Edward Horton
George W. James
Merritt S. Jones
David R. Johnston
Charles Kerr
G. F. Keiper
W. J. Kelsey
John L. Kite
Charles E. Keuster
C. E Lamon
J. H. Leal
Josiah Lee
J. G. Meachem, Jr.
L. B. Morrow
William A. Morse
G. D. Maxson
William M. Newell
N. W. Nesmeth
Joseph Otto
William P. Penfield
John W. Powell
Joseph L. Prentiss
G. W. Priest
Charles H. Quinlan
Lafayette Redmon
A. J. Rodman
C. B. Reed
Flavel Shurtleff
J. L. Shepard
Emery Sherman, Jr.
Asbury E. Smith
W. H. H. Smith
M. S. Stahl
G. A. Stevenson
D. Hedrick Stratton
G. C. Smythe
J. L. Trousdale
John W. Trueworthy
Henry Van Buren
G. W. Van Zant
Theodore Wild
Joseph H. Wilson

A. J. Darrah
S. A. Davison
S. W. Dodd
A. C. Douglass
A. S. Ehle
C. J. Lewis
A. W. Lueck
Carl J. Lucas
W. B. Lyons
Isaac L. Mahan
Horatio B. Withers
George Worsely
O. P. B. Wright
Charles Young

Martin Baker, M.D., W. H. Dubler, M.D., D. W. C. Denny, M.D., N. Wright, M.D., *ad eundem.*

1865–66.

Ethan P. Allen
T. E. Annis
S. B. Ayres
C. Isham Allen
Wm. J. Asdale
Luther Brown, Jr.
George W. Brown
T. Newton Booe
Edward E. Berry
George A. Clarke
Samuel C. Cravens
J. N. Crawford
James Cozad
John W. Craig
Richard Carscadden
Robert H. Crowder
James A. Comstock
George M. Chamberlin
Wm. J. Carter
James C. Davis
Franklin M. Denny
F. A. Dietrich
Jos. B. Eversole
Jerome B. Egbert
John A. Edmiston
Henry R. Fowler
J. C. Fitch
Chester S. Ford
John Guerin
W. L. Goodell
W. B. Graham
John N. Grover
C. Judson Gill
James E. Gowan
W. S. Goodell
John W. Grœsbeck, Jr.
Julius C. Holmes
Wm. J. Harris
Wm. Harper
Wm. S. Herrick
Carter B. Higgins
Abijah F. Henry
J. M. Hayward
Fred. W. Hoffman
E. Howard Irwin
Wm. H. H. King
George W. Langfitt
G. F. Lyons
Truman F. Loop
Peter T. Lange
Jacob W. Magelssen
James J. Morgan
James M. McMasters
A. Wilbur Meachem
John G. Munsell
W. W. Murray
S. C. Maxwell
Wm. D. Morehouse
E. A. Morse
John R. McDowell
Horace Nichols
S. F. Paddock
N. T. Quales
Rolla T. Richards
James J. Reed
Charles E. Rice
Wm. D. Rutledge
E. Malden Smith
M. P. Sigworth
Wm. D. Scott
D. Q Scheppers
M. F. Smith
Abram A. Sulcer
James E. Sutton
Charles E. Stedman
Charles True
Norman Teal
J. M. Taggart
Henry Tombœken
S. S. Troy
F. J. Van Vorhis
John T. Wilson
Robert L. Walston
Charles J. Winzenried
L. O. P. Wolfe
Francis W. Watson
George A. Wilson
R. B. Wetmore
M. V. B. Witherspoon
A. J. Willing

Albert H. Hoy, M.D., J. J. Brown, M.D., W. Louis Rabe, M.D., W. Y. Leonard, M.D., *ad eundem.*
Gerhard Christian Paoli, M.D., *Honorary.*

1866–67.

Curtis B. Ames
Upton A. Ager
William H. Buckmaster
Benjamin F. Brown
Charles C. Brown
Horatio N. Bradshaw
Robert J. Brackenridge
Gideon V. Bachelle
Otto Bosco
Wesley Clarke
Jerome H. Crouse
J. Gilbert Conner
Andrew P. Davis
Samuel Hawley
Semun R. Hewitt
John P. Humes
John N. Jones
Hiram D. Kellogg
Benjamin F. Kierulff
Justin Worthing Lamson
William A. Laflen
William J. Langfitt
Albert Morrall
Nicholas R. Marshall
Joseph K. Mayo
George E. Miller
George W. Ray
Charles A. Rockwood
Dolphus S. Randall
Jefferson Robinson
Stephen E. Robinson
Dan. S. Root
T. William Schwan
T. Newton Stewart
Irving R. Spooner
John Simpson
David T. Sellards
Lyman T. Strother
James K. Secord

| | | |
|---|---|---|
| William P. Dunne | Joseph W. Morey | Nelson L. Sweetland |
| William T. Dougan | William H. C. Moore | Samuel Thompson |
| Leonard W. Estabrook | Allen P. Mitten | John J. Taylor |
| William Eaton | Jerome C. Merrick | Alexander W. Trout |
| Charles A. Edgar | John Massmann | John C. Tatman |
| Curtis Treat Fenn | Frederick D. Morse | Thomas J. Tennery |
| James Luther Gandy | Alexander B. Newton | Henry B. Upton |
| Edward B. Hobson | Henby B. Newell | W. Hendrix Veatch |
| John Hughes | Sanford T. Odell | Evart Van Buren, Jr. |
| Wm. Baker Hathaway | Henry K. Palmer | Joseph Van Cowan |
| John W. Hensley | William Porter | |

James Murphy, M.D., Maximillian A. Cachot, M.D, *ad eundem.*
David Prince, M.D., Ezra S. Carr, M.D., *Honorary.*

1867–68.

| | | |
|---|---|---|
| Francis G. Arter | William H. H. Hagey | William R. Page |
| James B. Armstrong | Byron Holmes | Joseph B. Rood |
| James H. Barnwell | Christian B. Hirsch | J. Rodney Rundlett |
| Hugh Brownlee | J. Robert Haggard | Wilhelm Rienholdt |
| James Barr | Walter L. Johnson | Antinous A. Rowley |
| A. W. Bosworth | Thomas C. Kimball | Wm. S. Robertson |
| James R. Barnett | Thomas N. Livesay | Justin Ross |
| James H. Baker | Gershom J. R. Little | William S. Rowley |
| Amos Babcock | Edmund L. Lathrop | E. H. Pardu |
| Robert N. Barger | William A. Looney | John G. Riddler |
| William H. Christie | Louis B. La Count | Corydon Richmond |
| Pascal L. Craig | John G. McKinney | Royal Reed |
| John Cassidy | Abraham Miller | Harrion Stelle |
| Henry A. Chase | Ben. C. Miller | Ebert S. Sharon |
| James M. Cook | Charles Muth | Daniel Spittler |
| J. A. Carter | Leonidas B. Martin | Josiah T. Scovill |
| F. Wallace Coffin | James McClure | John W. Shiption |
| John B. Draper | John B. Moore | DeWitt Clinton Smith |
| Nelson A. Drake | Americus V Moore | S. E. Scanland |
| David L. Davidson | Samuel P. McCrea | John P Seawright |
| Thomas A. Elder | Thomas C. McCoughey | Oscar F. Seeley |
| George W. Elkins | William J. Maynard | John F. Shronts |
| John T. Foster | Thomas C. Murphy | Dana B. Segur |
| John G. Frank | Erancis McGuire | Charles B. Thrall |
| Benjamin H. Freeland | Charles A. McCollum | D. H. Arthur Thrane |
| David M. Finley | Albert B. McKune | George O. Taylor |
| Frank Fifield | Edmund L. Mayo, Jr. | John E. Tuttle |
| William Flinn | James Moffit | L. E. Towne |
| William J. Fern | Albertis P. McCullock | W. Alphonso Wood |
| John A. M. Gibbs | William R. McMahan | D. Lindley Woods |
| Lyman T. Goodner | Garrett Newkirk | Matthias S. Wheeler |
| John H. Goodell | John R. O'Reiley | Thomas Audley Wakely |
| John B. Griswold | Charles T. Parkes | Charles A. Wheaton |
| Henry C. Gemmell | William Quivey | Richard M. Wigginton |
| Samuel R. Hicks | William S. Pitts | Hiram G. Wyckoff |
| Abrogene Holland | Joel Prescott | Rush Winslow |
| Cyrus Heywood | John H. Feters | James I. Wakefield |
| Fernand Henrotin | Bennett A. Payne | Henry Joseph Warworth |
| Merritt Hurst | James Pankhurst | Thomas J. Yount |

Daniel C. Babcock, John W. Cowden, W. F. Hani, William Little, William N. Bailey, Abram Hard, } *ad eundem.*

Joseph Van Dyke, Orpheus Everts, John Ten Broek, J. J. Woodward, J. S. Bobbs, } *Honorary.*

### CATALOGUE OF STUDENTS,

### 1867-1868.

Francis G. Arter, Illinois.
Wm. F. Artz, Illinois.
James Armstrong, Illinois.
Wm. J. Ashby, Indiana.
Horace J. Avery, M.D., Michigan.
Arthur B. Brackett, Illinois.
Hugh Brown, Iowa.
Wm. M. Boyd, Illinois.
James H. Baker, Indiana
Robert Briggs, Illinois.
Amos Babcock, Iowa.
James Barr, Iowa.
Robert M. Barger, Illinois.
Wm. G. Bradley, Illinois.
Russell Broughton, Wisconsin.
James R. Barnett, Wisconsin.
John M. Bowers, M.D., Illinois,
Hugh Brownlee, Indiana.
Simon P. Brown, Illinois.
D. J. Brookings, Illinois.
F. W. Belfield, Illinois.
James H. Baker, Missouri.
James H. Barnwell, Iowa.
Daniel C. Babcock, M.D., Wisconsin.
Wm. N. Bailey, M.D., Indiana.
A. W. Bosworth, Illinois.
T. D. Brown, Wisconsin.
Wm. H. Christie, Illinois.
John Cassidy, Indiana.
John J Carlin, Illinois.
James A. Carter, Illinois.
James M. Cook, Michigan
Stephen H. Clizbe, Michigan.
Henry P. Crawford, Illinois.
Wm. G. Cochran, Illinois.
Pascal L. Craig, Wisconsin.
John P. Cowdin, Illinois.
James W. Campbell, Missouri.
F. Wallace Coffin, Ohio.
Thomas Cosgrove, Wisconsin.
Henry A. Chase, Wisconsin.
Nelson H. Church, Indiana.
Mauritz B Carleman, Illinois.
R. W. Cavins, Indiana.
Cass Chenoweth, Illinois.
Peter A. Collet, Iowa.
James H. Callahan, Illinois.
John L. Conelly, Illinois.
John W. Cowden, M.D., Iowa.
P. Curran, Illinois.
David E. Cutler, Iowa.
John A. Cook, M.D., Illinois.
Orestes A. Crossman, Michigan.
David L. Davidson, Illinois.
Nelson A. Drake, Wisconsin.
Samuel W. Durant, Illinois.
Albert A. Dye, Wisconsin.
Michael Donnelly, Minnesota.
John B. Draper, Missouri.
John T. Dake, Iowa.
William Dougall, Indiana.
John M. Dod, Illinois.
John Dancer, M.D., Indiana.
Jas. M. Dougheny, M.D., Kentucky.
P. W. Easling, Iowa.
Thomas A. Elder, Pennsylvania.
George W. Elkins, Illinois.
Arthur W. Edwards, Wisconsin.
William Everett, Illinois.
Perry N. Evans, Illinois.
Gustav Fricke, Illinois.
William Flinn, Wisconsin.
John T. Foster, Illinois.
David M. Finley Iowa.
Nelson P. Frost, Illinois.
Wm. J. Fern, Illinois.
John J. Fisher, Indiana.
George W. Farrow, Illinois.
Ezra K. Friermood, Indiana.
Frank Fifield, Illinois.
Wilson T. Freeman, Indiana.
David B. Fonda, Illinois.
Benjamin H. Freeland, Indiana.
John G. Frank, Illincis.
J. Robinson Groesbeck, New York.
George Green, Illinois.
John Green, Illinois.
Lyman T. Goodner, Illinois.
Frederic R. Goodwin, Illinois.
Charles W. Goodale, Indiana.
William A. Gordon, Wisconsin.
Henry C. Gemmill, Indiana.
Joseph C. Gifford, Indiana.
Joseph B. Griswold, Minnesota.

Hugh C. Graham, Illinois.
James F. Gower, Indiana.
Albert E. Gibbs, Illinois.
Septimus W. Gould, Illinois.
John H. Goodell. Illinois.
John W. Goe, Wisconsin.
Leslie Gillett, M.D., Illinois.
Samuel M. Green, Indiana.
John A. M. Gibbs, Illinois.
Julius T. C. Hoffman, Illinois.
Christian J. B. Hirsch, Illinois.
Byron Holmes, Wisconsin.
Fernand Henrotin, Illinois.
Geo. W. Hudson, Illinois.
J. Robert Haggard, Illinois.
James H. Hutchins, Illinois.
Chas. E. Hogeboom, Illinois.
John B. Hamilton, Illinois.
George C. Hall, Wisconsin.
James R. Holgate, Illinois.
Samuel K. Hicks, California.
Robert M. Hollingsworth, Indiana.
Wm. H. H. Hagey, Illinois.
Chas. C. Hamrick Indiana.
Wm. C. Hoover, Indiana.
Cyrus Haywood, Illinois.
James Henry, Iowa.
John A. Henry, Iowa.
F. C. Hageman, Illinois.
Thomas J. Harvey, Illinois.
Ambrogene Holland, Iowa.
Ulrick Heyerdahl, Illinois.
Merritt Hurst, Illinois.
Wm. F Hani, M.D., Indiana.
H. W. Hart, M.D., Iowa.
Rev. Thomas C. Hood, Tennessee.
Peregrine C. Jones, Illinois.
Walter L. Johnson, Indiana.
Howard L. Johnson, M.D., Kentucky.
Jahiel C. Kilgore, Illinois.
Thomas C. Kimball, Indiana.
Anders Klingberg, Illinois.
Raymond L. Leonard, Illinois.
Thomas N. Livesey, Illinois.
Joseph R. Laine, Wisconsin.
Gershon J. Little, Illinois.
Hugh E. Lindsay, Illinois.
Justin J. Leavitt, Wisconsin.
W. Henry Leis, Illinois.
George W. Lee, Wisconsin.
Eli J. Lemon, Illinois.
F. H. Linde, Wisconsin.
William A. Looney, Illlnois.
Louis B. LaCourt, Wisconsin.
Edmund L. Lathrop, Illinois.
S. W. Lee, Illinois.
Andrew L. Logan, M.D., Pennsylvania.
Ben. C. Miller, Indiana.
A. Siedschlag Mansfelde, Illinois.
Alburtis P. McCullock, Iowa.
Albert B. McKune, Illinois.
John A. Mandeville, Illinois.
Isaac H. McCoy, Iowa.
Edward L. Mayo, Illinois.
Chas. C. Merrill, Illinois.
Wm. R. McMahan, Missouri.
John M. Morse, Illinois.
Wm. L. McLane, Illinois.
Abraham Miller, Indiana.
Charles Muth, Wisconsin.
L. B. Martin, Illinois
John B. Moore, Indiana.
Adam E. Miller, Illinois.
Alexander McKean, Iowa.
Chas. A. McCollom, Minnesota.
Albert B. Modesitt, Indiana.
Thomas C. McCaughey, Illinois.
Samuel P. McCrea, Indiana.
Americus V Moore, Indiana.
John G. McKinney, Illinois.
John McGinnis, Illinois.
Wm. J. Maynard. Michigan.
Thomas C. Murphy, Illinois.
James McClure, Indiana.
James Moffltt, Indiana.
Arthur G. Murphy, Illinois.
Otis Moore Illinois.
James T. Moffat, Indiana.
Francis McGuire, Wisconsin.
John McLean, M D., Illinois.
John J. McDonnell, Illinois.
John W. McKinney, Illinois.
Wm. Mansen, M.D., Kansas.
E. B. Marshall, M.D., Illinois.
Wm. S. Mattocks, Canada West.
Caleb Nanscawen, Wisconsin.
Garrett Newkirk, Illinois.
George B. Noyes, Illinois.
Samuel J. Nurheimer, Indiana.
Chas. T. Parks, Illinois
Joel Prescott, Illinois.
Wm. L. Pitts, Iowa.
James Parkhurst, Illinois.
Wm. R. Page, Illinois.
Robert O. Purviance, Illinois.
J. H. Phelps, Illinois.
John H. Peters, Illinois.
Allen Payne, Illinois.
Arthur T. Poysius, M.D., Michigan.
Joseph B. Rood, Illinois.
John B. Ralph, Illinois.
Robert N. Rickey, Illinois.
Wm. S. Robertson, Indiana.
Chas. W. Russell, Indiana.
John A. Ross, Alabama.
James W. Reeder, Illinois.

James R. Rundlett, Wisconsin.
Harley G. Ristine, Iowa.
Wilhelm Reinhold, Illinois.
Justin Ross, Indiana.
Royal Reed, Illinois.
John R. Reil'y, Michigan.
Antinous A. Rowley, Wisconsin.
James S. Rundell, M.D., Iowa.
John G. Riddler, Missouri.
Fred. P. Sovereign, Indiana.
Sylvester S. Smith, Canada West.
John P. Seawright, Indiana.
Dewitt C. Smith, Illinois.
Leander A. Sheetz, Illinois.
Frank D. Stannard, Illinois.
John F. Shronts, Illinois.
John W. Shipton, Illinois.
Wm. H. Shrock, Indiana.
George E. Stevens, Illinois.
Jacob A. Smith, Illinois.
Henry H. Sloan, Illinois.
Josiah T. Scovill, Michigan.
Joseph H. Smith, Illinois.
Henry C. Soule, Wisconsin.
Ebert A. Sharon, Illinois.
Dana B. Segar, Illinois.
Wm. E. Sloat, Wisconsin.
S. E. Scanland, Illinois.
Daniel Spitler, Indiana.
Oscar F Seeley, Michigan.
George P. Sullivan, Illinois.
John Simpson, M.D., Illinois.
Harrison Steele, Illinois.
Byron Stevens, Wisconsin.
Arthur Thrane, Illinois.
L. E. Towne, Wisconsin.
Albert R. Tucker, Indiana.
Chas B. Thrall, Wisconsin.
D. G. M. Trous, Indiana.
Wm. Tisor, Indiana.
John E. Tuttle, Illinois.
Oscar Taylor, Illinois.
George O. Taylor, Kentucky.
Horace J Truax, M.D., Nebraska.
Claude C. Underwood, Illinois.
Wm. L. Underwood, Illinois.
Franz Unsticker, M.D., Ohio.
W. Alphonso Wood, Indiana.
A. C. Williams, Illinois.
John Williamson, Illinois.
Chas. A. Wheaton, Wisconsin.
Basil M. Webster, Iowa.
Thos. A. Wakely, Illinois.
Robert M. Wilson, Illinois.
Wm. H. Wirt, Ohio.
Henry A. Winter, Illinois.
Edward N. Wheeler, Illinois.
Otho B. Will, Illinois.
George H. Waller, Illinois.
Chas. A. White, Indiana.
Richard M. Wigginton, Wisconsin.
John C. Webster, Indiana.
Jacob K. Wagner, Iowa.
John L. Whitley, Iowa.
George Williamson, Illinois.
Chas. E. Walker, Illinois.
J. B. Walker, M.D., Illinois.
Daniel W. Warren, Illinois.
Hiram G. Wyckoff, Illinois.
Rush Winslow, Wisconsin.
Matthew S. Wheeler, Illinois.
Daniel L. Woods, Indiana.
John S. Watson, Illinois.
J. I. Wakefield, Iowa.
Frank L. Wadsworth, Illinois.
H. J. Warmuth, Tennessee.
E. C. Wilder, Illinois.
Thomas J. Yount, Iowa.
James W. Young, New York.
Edward J. Young, Missouri.

## Figures and Tables

**Figure f1:**